# Editorial: Microfluidics and mass spectrometry in drug discovery and development: from synthesis to evaluation

**DOI:** 10.3389/fphar.2023.1201926

**Published:** 2023-05-19

**Authors:** Giuseppe Caruso

**Affiliations:** ^1^ Departmento of Drug and Health Sciences, University of Catania, Catania, Italy; ^2^ Unit of Neuropharmacology and Translational Neurosciences, Oasi Research Institute-IRCCS, Troina, Italy

**Keywords:** 3D tumor model, large-scale validation, molecule generation, machine learning, extractive electrospray ionization mass spectrometry, microfluidics, carnosine, drug discovery

Because of the current unmet medical needs, new drugs are urgently required. In this context, the activities related to drug discovery and development play a key role, allowing the delivery of new drugs to the market, and thus available at the pharmacy shelf (Vohora and Singh, 2017). Traditionally, with regards to drug discovery, the main source of new drug entities was represented by natural molecules, but during the last decades there was a shift toward high-throughput synthesis and combinatorial chemistry-based development (Alhakamy et al., 2020; Berdigaliyev and Aljofan, 2020). The aim of the “discovery phase,” including numerous processes such as target identification and validation, is the identification of a drug candidate for further development, while the “development phase” relates to the optimization of chemical synthesis, the formulation as well as the preclinical toxicological studies, the clinical trials, and, in the best scenario, the regulatory approval. Both phases are very expensive and time-consuming, and most of the time, despite the identification of thousands of drug candidates, none or very few of them will be approved. Studies considering all therapeutic areas indicate that the development of a new drug will take an average of 10–15 years (or even longer) (Mohs and Greig, 2017), reason why new tools and innovative approaches are urgently needed to guide new drug development.

Microfluidics represents one of the most promising tools for this research compared to conventional drug discovery and development processes, allowing, among other things, system miniaturization, small sample consumption, and a highly controlled environment for drug synthesis and drug delivery system fabrication (Caruso et al., 2020). Microfluidic technologies also offer the ability to mimic both physiological and pathological conditions observed *in vivo*, and, very importantly, are compatible with several analytical strategies for high throughput drug screening and evaluations, including mass spectrometry (MS) and high-performance liquid chromatography (HPLC), two well-established methods employed in drug discovery and development (Wilson, 2011).

There are three original research and one review articles in this Research Topic.

The first study, carried out by Xiao et al. focused on the establishment of a matrigel-based 3D micro-tumor model on an array chip for large-scale anticancer drug evaluation. The effects of numerous chemotherapeutics against cancer cell lines was investigated demonstrating a higher drug resistance in the case of the 3D model. The latter also showed a higher specificity to targeted drugs compared with the corresponding 2D model. Finally, the evaluation results on the 3D tumor model were more consistent with the *in vivo* cell-derived xenograft model, allowing to exclude 95% false-positive results coming from the 2D model. The matrigel-based 3D micro-tumor model on an array chip developed by the authors represents a promising tool to speed up cancer drug discovery process.

Molecular generation through machine learning has speeded the structural optimization of drugs, especially in the case of targets for which a large amount of bioactivity data has been reported, while is, unfortunately, often powerless when considering new targets (Walters and Barzilay, 2021; von Korff and Sander, 2022). The second original research article of Xiong et al. represents the first study in which DNA-encoded library (DEL)’s dataset was used instead of public databases for molecular generation, allowing to overcome the above limitation. DEL data-based molecular generation could represent a powerful tool allowing not only the improvement of drug discovery but also the enhancement of structural optimization technique.

The last original research contribution belongs to Privitera et al., describing the coupling of microfluidics to HPLC to study the protective activity exerted by carnosine on human microglia challenged with lipopolysaccharides (LPS) and ATP, an *in vitro* model mimicking the molecular alterations observed in depressed patients (e.g., oxidative stress and energy metabolism impairment). Microglia represent a common model of LPS-induced activation to identify novel pharmacological targets for depression and Alzheimer’s disease, and numerous studies have linked the impairment of energy metabolism to the onset of depressive episodes (Park et al., 2015). The *in vitro* model characterized by the authors as well as the combination of the above-mentioned techniques could be of great importance, giving perspectives for the development of innovative therapeutic strategies in the context of systemic and neurodegenerative disorders.

The review by Qin et al. describes the application of extractive electrospray ionization mass spectrometry (EESI-MS) for analytical evaluation and synthetic preparation of pharmaceuticals. EESI-MS allows the effective ionization of molecules of interest, even if at low concentration, in complex mixtures, with high sensitivity, selectivity, and responding speed, with no need of any sample pretreatment. In addition to that, this technique makes it possible at high-energy molecular species to undergo specific reactions leading to advanced functionalization. As highlighted by the authors, EESI-MS could be further developed in several aspects, including *in vivo* analysis and reliability, thus providing a unique platform for wide applications in numerous disciplines such as chemistry, biology, and the life sciences (Chen et al., 2007).

In conclusion, all the articles published covered different and current aspects related to the proposed Research Topic, considering the importance of microfluidics and/or mass spectrometry in drug discovery and development processes. The combination of *in vitro* and *in vivo* preclinical studies with *in silico* ones is of utmost importance to increase the findings related to drug discovery and development and to translate them to clinical practice.
